# Lactic acid bacteria as starter cultures: An update in their metabolism and genetics

**DOI:** 10.3934/microbiol.2018.4.665

**Published:** 2018-12-11

**Authors:** Thomas Bintsis

**Affiliations:** Department of Agricultural Technology, TEI of West Macedonia, 53100 Florina, Greece

**Keywords:** lactic acid bacteria, genetics, applications, fermented food

## Abstract

Lactic acid bacteria (LAB) are members of an heterogenous group of bacteria which plays a significant role in a variety of fermentation processes. The general description of the bacteria included in the group is gram-positive, non-sporing, non-respiring cocci or rods. An overview of the genetics of lactococci, *Streptococcus thermophilus*, lactobacilli, pediococci, leuconostocs, enterococci and oenococciis presented with special reference to their metabolic traits. The three main pathways in which LAB are involved in the manufacture of fermented foods and the development of their flavour, are (a) glycolysis (fermentation of sugars), (b) lipolysis (degradation of fat) and (c) proteolysis (degradation of proteins). Although the major metabolic action is the production of lactic acid from the fermentation of carbohydrates, that is, the acidification of the food, LAB are involved in the production of many beneficial compounds such as organic acids, polyols, exopolysaccharides and antimicrobial compounds, and thus have a great number of applications in the food industry (i.e. starter cultures). With the advances in the genetics, molecular biology, physiology, and biochemistry and the reveal and publication of the complete genome sequence of a great number of LAB, new insights and applications for these bacteria have appeared and a variety of commercial starter, functional, bio-protective and probiotic cultures with desirable properties have marketed.

## 1. Introduction

Lactic acid bacteria (LAB) play a multifunctional role in food, agricultural, and clinical applications [1]. Using LAB in food fermentation is one of the ancient known food preserving techniques. Fermented milk products, such as yogurt and cheese, appeared in human diet about 8,000–10,000 years ago. Up to the 20th century, food fermentation remained an unregulated process, and, the discovery and characterization of LAB have changed the views on food fermentation.

Properties such as nutritional, environmental, and adhesional adaptations have provided LAB with the ability to adapt and present in different environments ranging from food matrices such as dairy products, meats, vegetables, sourdough bread. In addition, LAB are common inhabitants of human mucosal surfaces such as oral cavity, vagina, and gastrointestinal tract [2]. Metabolic activities are associated with production of many beneficial compounds such as organic acids, polyols, exopolysaccharides and antimicrobial compounds [1],[3].

The traditional method for the manufacture of fermented food products was the “inoculation” of the food with a sample of a previous day product, i.e. back-slopping. This method has certain drawbacks, mainly a great fluctuation in the quality of the product, but is still used for some home-made products. The substitution of the back-slopping with a selected starter culture was very early realized to be a necessity. Nowadays, since the production of fermented foods is automated and produced in large quantities with total control of the process, the use of commercial starter cultures is an integral part of a successful production of any fermented product. Starters are divided into defined- and mixed-strain cultures. Defined-strain cultures are pure cultures with known physiological characteristics and technological properties. These consist of 2–6 strains, used in rotation as paired single strains or as multiple strains and enable industrial-scale production of high quality products. Mixed-strain cultures contain unknown numbers of strains of the same species and may also contain bacteria from different species or genera of LAB [4]. For a detailed classification of starter cultures see [4]–[8].

An authoritative list of microorganisms with a documented use in food was established as a result of a joint project between the International Dairy Federation (IDF) and the European Food and Feed Cultures Association (EFFCA) and recommended for Qualified Presumption of Safety (QPS list) [9]. The 2012 IDF-EFFCA inventory contains 195 bacterial species and 69 species of yeasts and moulds [10] and the updated list of 2017 reconfirmed their status for all LAB included in the list as well their qualifications [11].

LAB are classified as Gram-positive bacteria which include low Guanine + Cytosine (G + C) content as well as being acid tolerant, non-motile, non-spore forming and are rod- or cocci-shaped. The main function of LAB is to produce lactic acid, that is, the acidification of the food. Thus, the main application of LAB is as starter cultures in the food industry with an enormous variety of fermented dairy products, meat, fish, fruit, vegetable and cereal products. Besides, LAB contribute to the flavour, texture and nutritional value of the fermented foods, through production of aroma components, and used as adjunct cultures [12],[13]; production or degradation of exopolysaccharides, lipids and proteins, production of nutritional components such as vitamins, and used as functional cultures, and promoting therapeutic effects and used as probiotics [1],[13],[14]. In addition, they contribute to the inhibition of spoilage and pathogenic microorganisms and thus, used as bio-protective cultures [15].

## 2. Metabolism of LAB

The three main pathways which are involved in the development of flavour in fermented food products are glycolysis (fermentation of sugars), lipolysis (degradation of fat) and proteolysis (degradation of proteins) [16]–[18]. Lactate is the main product generated from the metabolism of lactose and a fraction of the intermediate pyruvate can alternatively be converted to diacetyl, acetoin, acetaldehyde or acetic acid (some of which can be important for typical yogurt flavours). The contribution of LAB to lipolysis is relatively little, but proteolysis is the key biochemical pathway for the development of flavour in fermented foods [16]–[19]. Degradation of proteins by the activities of rennet enzymes and the cell-envelope proteinase and peptidases yields small peptides and free amino acids, the latter of which can be further converted to various alcohols, aldehydes, acids, esters and sulphur compounds for specific flavour development in dairy products [16],[20].

### 2.1. Glucose metabolism

**Figure 1.**
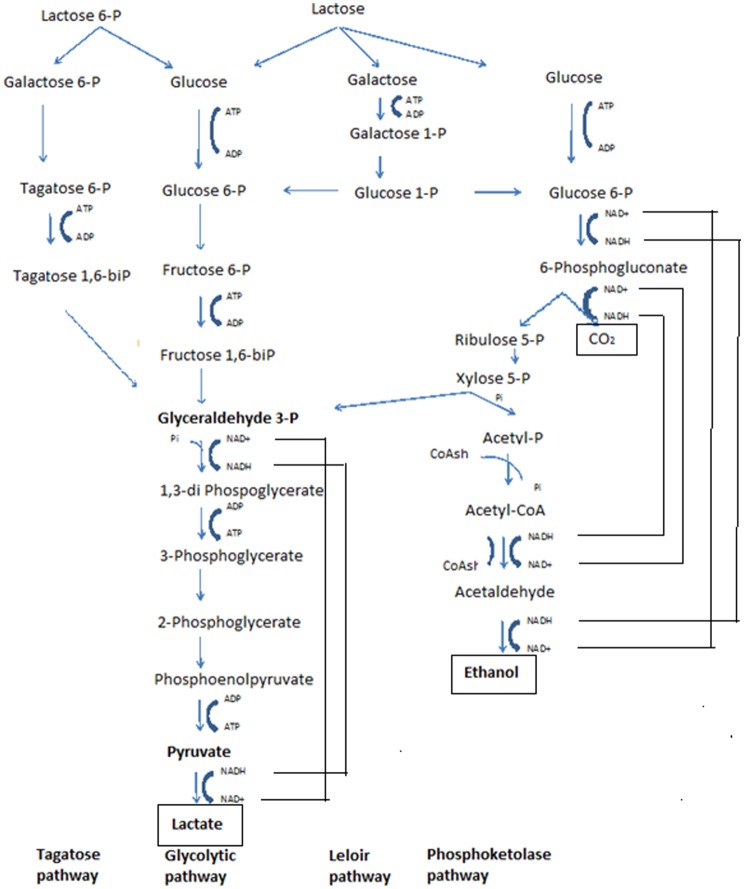
Lactose metabolism pathways in lactic acid bacteria. After: [4],[16]–[18].

LAB need a sugar for energy production and subsequent growth. Fermentation of lactose is called glycolysis or glycolytic pathway (Figure 1). Obligatory homo-fermentative LAB are those that ferment lactose into pyruvic acid, which is then reduced to lactic acid by the reducing power previously produced in the form of NADH. Thus, lactic acid is obtained as the sole product (Glucose gives 2 Lactic Acid and 2 ATP moles) and this process is called homo-lactic fermentation [18]. Obligatory homo-fermentative LAB include, among others, *Lactobacillus acidophilus*, *Lactobacillus amylophilus*, *Lactobacillus bulgaricus* and *Lactobacillus helveticus* [18]. Homo-lactic fermentation should theoretically yield 2 moles of lactic acid per mole of consumed glucose with a theoretical yield of 1 g of product per g of substrate, but the experimental yields are usually lower (0.74–0.99 g/g) because a portion of the carbon source is used for biomass production (0.07–0.22 g/g) [18],[19]. Under stress conditions such as carbon source limitation, presence of different carbon sources other than glucose, high pH or low temperature, some homo-fermentative microorganisms can produce formic acid by mixed acid fermentation [20] by the action of pyruvate-formate lyase [21].

Hetero-lactic fermentation is the process that is characterized by the formation of co-products such as CO_2_, ethanol and/or acetic acid in addition to lactic acid as the end product of fermentation—phosphoketolase pathway (Figure 1). The first step of glucose degradation, which is called pentose phosphate pathway, leads to glyceraldehyde 3-phosphate, acetyl-phosphate and CO_2_. Glyceraldehyde 3-phosphate enters the glycolysis through which it is transformed into lactic acid, while acetyl-phosphate is converted into acetic acid and/or ethanol (Glucose gives Lactic acid and CO_2_ and Ethanol and ATP or Glucose gives Lactic acid and CO_2_ and Acetic acid and 2 ATP and 2 NADH). The relationship between the amounts of acetic acid and ethanol, which reduces the theoretical yield to 0.5 g/g, depends on the ability of the microorganism to reoxidize the NADH generated in the early stages of the process along with its energy requirements. Microorganisms that use only this metabolic pathway for the consumption of carbohydrates are called obligatory hetero-fermentative, among which are *Lactobacillus brevis*, *Lactobacillus fermentum* and *Lactobacillus reuteri* [18].

In addition to glucose, there are other hexoses such as fructose, mannose or galactose, which can be consumed by LAB [18]. On the other hand, hexose-fermenting lactobacilli are unable to ferment pentoses. There are some species of this genus, classified as facultative hetero-fermentative, among which *Lactobacillus alimentarius*, *Lactobacillus plantarum*, *Lactobacillus casei*, *Lactobacillus rhamnosus*, *Lactococcus lactis*, *Lactobacillus pentosus* and *Lactobacillus xylosus* [18], that perform both fermentations, consuming hexoses by the homo-lactic pathway and pentoses by the hetero-lactic one. The catabolism of pentoses requires additional conversion steps through which they are transformed into metabolic intermediates of the pentose phosphate pathway. By this way, as an instance, xylose is transformed into xylulose and then phosphorylated to xylulose 5-phosphate, arabinose into ribulose, and this in turn is phosphorylated to ribulose 5-phosphate [21].

**Figure 2.**
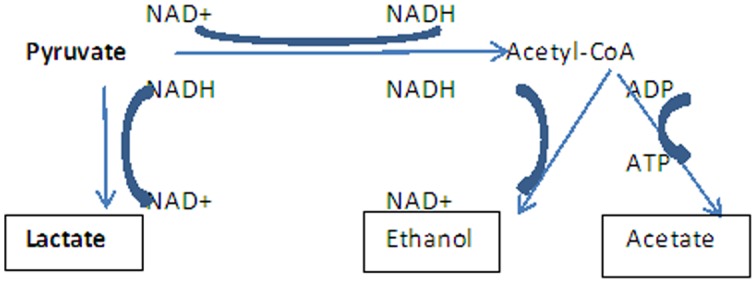
Pyruvate metabolism in lactic acid bacteria. After: [4],[16]–[18].

LAB can also metabolize disaccharides such as lactose, maltose and sucrose, which are cleaved by the action of endocellular hydrolases. Lactose fermentation by LAB has been reviewed by [15],[16],[21],[22]. After transporting into the cell, lactose is fermented with one of the four pathways as shown in Figure 1. For example, in lactococci the tagatose pathway is followed and lactose transport and the enzymes for the pathway are plasmid encoded [23]. Galactose is only metabolized by *Lb. helveticus* and some strains of *Lb. delbruecki* subsp. *lactis* (Gal+) and probably leuconstoc *via* Leloir pathway. Glucose-6-P is metabolized by the glucolytic pathway in the lactobacilli and by phosphoketolase pathway in leuconstoc. L-lactate is generally the sole product of fermentation, but when LAB are grown on galactose, maltose or low levels of glucose other product are formed, form pyruvate metabolism (Figure 2).

**Figure 3.**
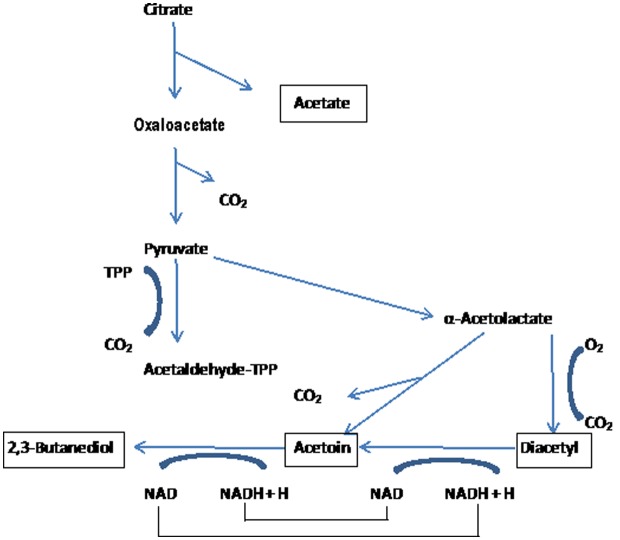
Citrate metabolism in lactic acid bacteria. After: [4],[16]–[18].

Citrate and malate are the most abundant organic acids in plants. Citrate metabolism is important in fermented dairy products, while malate metabolism is important in wine. The organisms responsible for citrate metabolism in starter cultures are leuconostoc and Cit+ lactococci. Citrate is hydrolyzed to oxaloacetate and acetate by citrate lyase. Citrate lyase is inducible in leuconostocs and constitutive in Cit+ lactococci [24]. The oxaloacetate is decarboxylated to pyruvate, which can undergo several further transformations to diacetyl, acetoin, and 2,3-butylene glycol. Citrate is metabolized by *Leuconostoc* spp. and some strains of *Lc. lactis* subsp. *lactis* (citrate-utilizing, Cit^+^) to CO_2_ which is responsible for “eye” formation in some cheeses [16]. In addition, other important aroma compounds are produced in fermented milks, cheese and butter (Figure 3). Cit^+^ strains of *Lc. lactis* subsp. *lactis* contain a plasmid which encodes the transport of citrate. Citrate metabolism has been reviewed by Hugenholtz [25]. The presence of a citrate permease is essential for the metabolism of citrate. The citrate permeases of both *Lc. lactis* subsp. *lactis* and *Leuconostoc* spp. were found to be pH dependant and their highest acidity was between pH 5.0 and 6.0. The citrate inside the cell is converted to oxaloacetate, by the enzyme citrate lyase, and then oxaloacetate is decarboxylated to pyruvate. In lactococci, pyruvate is then converted to acetate, diacetyl, acetoin, 2,3-butanediol and CO_2_. The enzyme pyruvate formate lyase is able to convert pyruvate to formate, acetate, acetaldehyde and ethanol under anaerobic conditions and at high pH (>7.0). Under aerobic conditions and at pH 5.5 to 6.5, pyruvate can be converted to acetate, acetaldehyde, ethanol and the minor products acetoin, diacetyl and 2,3-butanediol *via* the multi-enzyme pyruvate dehydrogenase complex (Figure 3). In *Leuconostoc* spp., the pyruvate produced from citrate is converted to lactate, although at low pH and in the absence of glucose (or lactose) *Leuconostoc* spp. will produce diacetyl and acetoin. Acetate is also formed *via* the hetero-fermentative metabolism of lactose during co-metabolism with citrate [22].

Malic acid is fermentable by LAB. Both homo-lactics and hetero-lactics are able to decarboxylate malic acid to lactic acid and CO_2_. Minimal CO_2_ production has been considered beneficial in maintaining anaerobiosis in sauerkraut. In cucumber fermentation, CO_2_ production causes bloater damage. The decarboxylation of malic acid is undesirable in cucumber fermentation. *Lb. plantarum* produces most of the CO_2_ during cucumber juice fermentation *via* the decarboxylation of malic acid [26]. Strains of *Lb. plantarum* that do not decarboxylate malic acid (MDC-) might improve cucumber fermentation. Some MDC-mutants have been obtained through N-methyl-NV-nitro-N-nitrosoguanidine mutagenesis of MDC+ parent strains. These mutants did not produce significant amounts of CO_2_ when they fermented cucumber juice containing native malate [27].

### 2.2. Starch metabolism

Although hydrolyzing starch to simple sugars is not important in traditional fermented vegetables, a few amylolytic LAB have been isolated from starchy raw materials. An investigation of Mexican pozol, a fermented maize dough, indicates that LAB accounted for 90–97% of the total active microflora [28]. During fermentation, the amylolytic LAB degrade the starch first, and then the resulting sugars allow a secondary flora to develop. An acidophilic starch hydrolyzing enzyme secreted from a strain of *Lb. plantarum* was isolated and partially purified. This enzyme has a molecular mass of approximately 230 kDa and is capable of hydrolyzing soluble starch, amylopectin, glycogen, and pullan. The major reaction products from soluble starch were maltotriose, maltotetraose, and maltopentaose. These reaction products suggest that this enzyme may hydrolyze both α-1,6- and α-1,4-glucosidic linkages [28].

### 2.3. Protein metabolism

LAB are fastidious microorganisms and are unable to synthesize many amino acids, vitamins and nucleic acid bases [14],[18],[21]. Depending on the species and the strain, LAB require from 6 to 14 different amino acids [27],[29]. Since free amino acids in milk are limited and amino acids are present as protein components, the growth of LAB requires the hydrolysis of milk proteins. The hydrolysis of peptides to free amino acids and the subsequent utilization of these amino acids is a central metabolic activity in LAB [14],[18], and proteolysis has been identified as the key process influencing the rate of flavour and texture development in most cheese varieties and has been reviewed [16],[17],[20] and the catabolism of amino acids has been reviewed by Kunji et al. [30]. The degradation of milk proteins to peptides is catalysed by proteolytic enzymes present in LAB [31],[32], and peptides are then further hydrolysed by exopeptidases and endopeptidases to small peptides and amino acids [32].

LAB have only weak proteolytic action on myofibrillar proteins in fermented meat products [19],[28]. However, some *Lb. casei*, *Lb. plantarum*, *Lb. curvatus* and *Lb. sakei* strains actively contribute to the hydrolysis of the sarcoplasmic proteins [33] and to the subsequent decomposition of peptides into amino acids. Several peptidase activities have been reported in *Lb. sakei*, *Lb. curvatus* and *Lb. plantarum* isolated from sausages [19]. Further, some *Lb. sakei*, *Lb. curvatus* and *Lb. plantarum* strains possess leucine and valine amino-peptidases, which contribute to the catabolism of proteins and peptides generating free amino acids, precursors of flavour compounds in the final product [34].

### 2.4. Lipid metabolism

The enzymatic metabolism of fat is limited during the manufacture of fermented food products. The degradation of milk fat releases free fatty acids and glycerol, monoacylglycerides or diacylglycerides. However, certain free fatty acids are essential flavour compounds in certain cheeses (e.g. caprine milk cheeses). In addition, they react with alcohols or free sulphydryl groups to form esters and thioesters, respectively, or act as precursors of a number of other favour compounds, such as lactones [34]. Esterase activity has been detected in various lactobacilli [35], and esters contribute to the characteristic flavour of Swiss-type [33],[34] and White-brined cheese [36].

## 3. Genetics

LAB used for starter cultures in fermented food products belong to a number of bacterial genera including *Lactococcus*, *Streptococcus*, *Lactobacillus*, *Pediococcus*, and *Leuconostoc*, all members of the *Firmicutes*. Moreover, some probiotic cultures are mostly members of the genus *Bifidobacterium*, which also produce lactic acid as one of their major fermentation end-products, however, from the taxonomical point of view, they are members of the *Actinobacteria*. In addition, *Enterococcus* spp. have been found to be part of many fermented food microbiota, but, since they may cause a number of infections and may acquire antibiotic resistance mechanisms, are not commonly used as starter cultures [37].

The genetics of the LAB used as starter cultures in the food industry have been reviewed [38]–[41] and an overview is presented below. In addition, complete genome sequences of a great number of LAB have been published [42].

### 3.1. Lactococcus spp.

Lactococci are mesophilic LAB that were first isolated from green plants [39]. These bacteria, previously designated as the lactic streptococci (*Streptococcus lactis* subsp. *lactis* or *S. lactis* subsp. *cremoris*) was placed in this new taxon in 1987 by Schleifer [39]. Lactococci are selected for use as starters based on their metabolic stability, their resistance to bacteriophage, and their ability to produce unique compounds—often from amino acid catabolism. *Lc. lactis* subsp. *lactis* form one of the main constituents in starter cultures where their most important role lies in their ability to produce acid in milk and to convert milk fat and protein into flavour compounds.

Eighty five *Lc. lactis* genomes have been completed up to now according to the data retrieved from [43]. Genome ranges in size from 2.3 to 2.7 Mb. The availability of these complete lists of genes allows drawing full metabolic pathways [44] and exploiting some interesting characteristics for the production of fermented foods. There are noticeable differences between strains, e.g. the chromosome of *Lc. lactis* subsp. *lactis* MG1363 is 160 kb larger than that of *Lc. lactis* subsp. *lactis* IL1403 and has an average G + C content of 35.8%, and thus, encodes more proteins [4].

*Lc. lactis* subsp. *cremoris* strains are preferred over *Lc. lactis* subsp. *lactis* strains because of their superior contribution to product flavor *via* unique metabolic mechanisms [45]. With the knowledge of the complete genome sequences, *Lc. lactis* subsp. *cremoris* was found to contain greater genome sizes than *Lc. lactis* subsp. *lactis* IL1403 (approximately 2.37 Mb), with *Lc. lactis* subsp. *cremoris* MG1363 containing the largest genome size of approximately 2.53 Mb, followed by *Lc. lactis* subsp. *cremoris* SK11 with a genome size of 2.44 Mb [42]. Interestingly, a complete set of competence genes was observed on the *Lc. lactis* subsp. *lactis* IL1403 genome, indicating that the strain may have the ability to undergo natural DNA transformation [41].

Many of the traits in lactococci which render these microorganisms suitable for dairy fermentations are in fact encoded on plasmids [43]. Traits such as lactose utilization, casein breakdown, bacteriophage resistance, bacteriocin production, antibiotic resistance, resistance to and transport of metal ions, and exopolysaccharide (EPS) production have all been associated with extra-chromosomal plasmid DNA. Plasmids isolated from lactococci range in size from 3 to 130 kb, have a G + C content of 30–40% and vary in function and distribution, with most strains carrying between 4 and 7 per cell [44]. Plasmids are commonly exchanged between strains *via* conjugation [43]–[46] and with the chromosome by insertion sequence (IS) elements [47]. Presumably, these exchanges and rearrangements mediate rapid strain adaptation and evolution but also add to the instability of important metabolic functions [48],[49].

### 3.2. Streptococcus thermoplilus

*Streptococcus thermoplilus* is the second most commercially important starter culture. *S. thermophilus* is used, along with *Lactobacillus* spp., as a starter culture for the manufacture of several important fermented dairy foods, including fermented milks, yogurt, Feta and Mozzarella cheeses [4]. Although research on the physiology of *S. thermophilus* has revealed important information on some of these properties, including sugar and protein metabolism, polysaccharide production, and flavor generation, and only recently has the genetic basis for many of these traits been determined.

According to the data retrieved from NCBI, 32 *S. thermophilus* genomes have been completed [42]. The genome of *S. thermophilus* is 1.8 Mb, making it among the smallest genomes of all LAB. Although a moderate thermophile, it is phylogenetically related to the more mesophilic lactococci and has a comparable low G + C ratio between 36.8 and 39%. Moreover, *S. thermophilus* is related to human pathogenic strains of streptococci such as *Streptococcus pneumoniae*, *Streptococcus pyogenes* and *Streptococcus agalactiae* [50]. However, the most important pathogenic determinants are either absent or present as pseudogenes, unless they encode basic cellular functions [50]. *S. thermophilus* has therefore diverged from its pathogenic relatives to occupy the well-defined ecological niche of milk [50]. Pastink et al. [51] compared, using a genome-scale metabolic model of *S. thermophilus* LMG18311 with those of *Lc. lactis* subsp. *lactis* and reported the minimal amino acid auxotrophy (only histidine and methionine or cysteine) of *S. thermophilus* and the broad range of volatiles produced by the strain compared to lactococci. The unique pathway for acetaldehyde production, which is responsible for yogurt flavour, was also identified in *S. thermophilus*.

Unlike *Lactococcus* spp., plasmids are thought to play a relatively insignificant role in *S. thermophilus*, reported to be found in about 20–59% of strains examined [52]–[54]. Streptococcal plasmids are generally small, ranging in size from 2.1 to 10 kb and encode few industrially useful phenotypic traits, which include low molecular weight, heat stress proteins and specificity subunits of bacteriophage-resistant restriction modification systems [55]–[57].

### 3.3. Lactobacillus spp.

The genus *Lactobacillus* encompasses a large number of different species that display a relatively large degree of diversity. Actually, it is the largest genus in the LAB group, with over fifty species in total. Similar to *S. thermophilus*, the lactobacilli also belong to the thermophilic group of LAB starter cultures.

The species *Lactobacillus delbrueckii* contain three subspecies, that is subsp. *delbrueckii*, subsp. *lactis* and subsp. *bulgaricus*. The 48 genome assemblies of *Lb. delbrueckii* subsp. *bulgaricus* has a median total length of 1.87624 Mb and a median protein count 1,641 and a median G + C% of 49.8 [42].

*Lb. plantarum* has one of the largest genomes known among LAB [58],[59]. The 294 genome assemblies of *Lb. plantarum* has a median total length of 3.2616 Mb and a median protein count 2,991 and a median G + C% of 44.4687 [42]. In addition, 224 complete sequences of plasmids have reported [42]. *Lb. plantarum* is the predominant microorganism in sourdough and many cereal-based fermented products, and is dominant due to its utilization of corn dextrins after the depletion of the fermentable sugars [1] and recently the antimicrobial and antifungal properties have investigated [60].

*Lactobacillus sanfranciscensis* is the predominant LAB in sourdough [1] and 14 genome assemblies of *Lb. sanfranciscensis* have been reported, which have a median total length of 1.28747 Mb and a median protein count 1,221 and a median G + C% of 34.7 [42].

*Lactobacillus paracasei* subsp. *paracasei* is frequently recovered from matured cheese and constitute, together with *Lb. plantarum*, *Lactobacillus curvatus*, *Lb. rhamnosus* and *Lactobacillus casei* the core microbiota of the non-starter LAB contributing to the maturation process [12],[13]. The 78 genome assemblies of *Lactobacillus paracasei* subsp. *paracasei* have been published. It has a median total length of 2.99174 Mb and a median protein count 2,899 and a median G + C% of 46.3 [42]. There are 36 plasmid annotation reports.

*Lb. rhamnosus* is one of the few species of *Lactobacillus* that have been used as probiotic organisms in functional foods. A strain of *Lb. rhamnosus*, designated HN001, has been identified that has both flavour enhancing and probiotic attributes, therefore, it can be used as an adjunct during cheese manufacture to reduce adventitious microflora, accelerate cheese ripening, and improve cheese flavor [31],[32].

*Lb. johnsonii* strains have been mainly isolated from the feces of humans and animals [61],[62], suggesting that these bacteria constitute part of the natural intestinal flora. The 10 complete genome sequences are available [42] and they have a median total length of 1.88 Mb and G + C 34.55%. *Lb. johnsonii* La1 (formerly *Lactobacillus acidophilus* La1) has been extensively studied for its probiotic properties and is commercialized in the LC1 fermented milk products [40]. La1 shows immunomodulatory properties [63],[64] and antimicrobial properties [65]–[67]. The 34 genome assemblies of *Lb. acidophilus* (used as probiotic) have been published. It has a median total length of 1.97643 Mb and a median protein count 1,866 and a median G + C% of 34.6 [42].

*Lactobacillus helveticus* is quite closely related (< 10% sequence divergence) to *Lb. amylovorus*, *Lb. acidophilus*, *Lb. delrueckii*, *Lb. acetotolerans*, *Lb. gasseri*, and *Lb. amylophilus* [68]. The 49 genome assemblies of *Lb. helveticus* has a median total length of 2.05581 Mb and a median protein count 1,720 and a median G + C% of 36.7 [42]. Approximately 40 chromosomal genes and four plasmids have been sequenced from *Lb. helveticus*. *Lb. helveticus* is a component of “thermophilic” starter cultures used in the manufacture of a number of fermented dairy products [4],[68] and grows on a relatively restricted number of carbohydrates that includes lactose and galactose and typically requires riboflavin, pantothenic acid and pyridoxal for growth [69],[70].

*Lactobacillus reuteri* is a member of the normal microbial community of the gut in humans and animals. This organism produces antibiotic compounds, such as reutericin and reuterin, which have inhibitory effects on pathogenic microorganisms. In addition, *Lb. reuteri* is commonly used as a probiotic to maintain the balance of the gut microbial flora and stimulate the intestinal immune system. The 117 genome assemblies of *Lb. reuteri* has a median total length of 2.16482 Mb and a median protein count 1,968 and a median G + C% of 38.6 [42].

*Lactobacillus sakei* subsp. s*akei* took its name from rice alcoholic beverage (i.e. sake), which was the product that it was first isolated. The 39 genome assemblies of *Lb. sakei* subsp. *sakei* has a median total length of 1.99426 Mb and a median protein count 1,893 and a median G + C% of 41.0381 [42].

*Lb. fermentum* can be found in many vegetable and cereal-based fermented foods and it has been extensively used as a probiotic. The 20 complete genome sequences have reported having median length of 1.99 Mb and G + C 51.85%. Recently, a probiotic from famous longevity villages in Korea from healthy adults who were aged above 80 years and had regular bowel movements were isolated [71]. The isolates showed strong binding to intestinal epithelial cells, high immune-enhancing activity, anti-inflammation activity, and anti-oxidation activity as well as high survival rates in the presence of artificial gastric juice and bile solution, that is all the characteristics for a promising probiotic culture.

### 3.4. Pediococcus spp.

Phylogenetically, *Pediococcus* and *Lactobacillus* are related and form a super-cluster; all species of *Pediococcus* fall within the *Lactobacillus casei*—*Pediococcus* sub-cluster. However, morphologically, they are distinct since they form tetrads *via* cell division in two perpendicular directions in a single plane. *Pediococcus* can be described as the only acidophilic, homo-fermentative, LAB that divide alternatively in two perpendicular directions to form tetrads [72]. *Pediococcus pentosaceus* can be isolated from a variety of plant materials and bacterial-ripened cheeses and is a typical component of the NSLAB of many cheese varieties during ripening [73] and has been suggested as an acid producing starter culture in the dairy fermentations [74],[75]. Strains of *P. pentosaceus* have been reported to contain between three and five resident plasmids [76]. Plasmid-linked traits include the ability to ferment raffinose, melibiose, and sucrose, as well as, the production of bacteriocins [77],[78]. Plasmids can be conjugally transferred between *Pediococcus* and *Enterococcus*, *Streptococcus*, or *Lactococcus* and electro-transformation has been utilized to introduce plasmids into pediococci, including *P. pentosaceus* [41]. *Pediococcus damnosus* (previously identified as *Pediococcus cerevisiae*), which is a homo-fermentative LAB species, together with *Lb. brevis*, are commonly found in limbic and gueuze beer [79]. These LAB are dominating the microfloras of beer production from 2 up to 10 months of fermentation/maturation process. They are, like some other LAB species, well adapted to the specific environment in beer, due to a plasmid-encoded transporter protein HorA and a multi-drug transporter ORF5, which transport the hop antimicrobial compounds out of the cytoplasm [79],[80]–[82]. Transcriptome analysis of a beer-spoiling *Lb. brevis* strain has indeed shown that plasmid transcription is important for growth in both gassed and degassed beers [83].

### 3.5. Leuconostoc spp.

*Leuconostoc mesenteroides* is a facultative anaerobe requiring complex growth factors and amino acids [84]. Most strains in liquid culture appear as cocci, occurring singly or in pairs and short chains; however, morphology can vary with growth conditions; cells grown in glucose or on solid media may have an elongated or rod-shaped morphology. Cells are Gram-positive, asporogenous and non-motile. The 16 complete genome sequences are available [42], having a median length of 1.9 Mb and G + C 37.75%. Although *Leuc. mesenteroides* is commonly found on fruits and vegetables, it has been extensively used as an industrial dairy starter culture [4]. Under micro-aerophilic conditions, has an hetero-fermentative reaction. Glucose and other hexose sugars are converted to equimolar amount of D-lactate, ethanol and CO_2_ *via* a combination of the hexose monophosphate and pentose phosphate pathways [85]. Other metabolic pathways include conversion of citrate to diacetyl and acetoin and production of dextrans and levan from sucrose [85].

### 3.6. Enterococcus spp.

Enterococci consists of organisms typically found in the intestines of mammals, although through fecal contamination they can appear in sewage, soil, and water. They cause a number of infections that are becoming increasingly a problem due to the number of antibiotic resistance mechanisms these organisms have picked up. Both *E. faecalis* and *E. faecium* cause similar diseases in humans, and are mainly distinguished by their metabolic capabilities. *E. faecium* is an opportunistic pathogen which causes a range of infections similar to those observed with *E. faecalis*, including urinary tract infections, bacteremia (bacteria in the blood), and infective endocarditis (inflammation of the membrane surrounding the heart) [86]–[88].

Enterococci possess a broad spectrum of antibiotic resistances and examples of such are vancomycin resistance in *E. gallinarum*, resistance towards streptogramins in *E. faecalis*, resistance to isoxazolylpenicillins, cephalosporins, monobactams, aminoglycosides, lincosamides and polymyxins [86]–[88].

### 3.7. Oenococcus spp.

*Oenococcus oeni* is another member of the LAB and it occurs naturally in marshes and similar environments. It carries out malolactic conversion during secondary fermentation in wine production which is the conversion of malic acid to lactic acid with a concomitant rise in pH, making the wine microbiologically stable and enhancing the sensory properties of the wine (aroma, flavor, and texture). This step occurs after the yeast has converted the sugars in the wine to ethanol and carbon dioxide. The organism's high tolerance to sulfite and ethanol mean that it will be the predominant organism in the wine at the end of fermentation where it cleans up the remaining sugars and converts the bitter-tasting malic acid [89]. *O. oeni* differentially encode several carbohydrate utilization and amino acid biosynthesis pathways which have resulted in adaptation to their individual ecological niches [89]. The 203 complete genome sequences have reported having median length of 1.91033 Mb and G + C 37.9% [42].

## 4. Genetic engineering

Over the last 15–20 years a number of attempts have been made to change metabolite production in LAB, *via* genetic engineering. Mainly, these attempts are focused on the production of other flavour compounds from lactic acid, usually by removing lactate dehydrogenase (LDH), the enzyme directly responsible for reduction of pyruvate to lactate [90],[91]. The relative simplicity of *Lc. lactis* subsp. *lactis* sugar metabolism *via* the pyruvate pathway (Figure 2) together with the availability of the complete genome sequence [4] makes this bacterium a great model for the study of LAB. The metabolic re-routing of sugar metabolism has been reviewed [92],[93]. Initial metabolic engineering of *Lc. lactis* has focused primarily on the re-routing of pyruvate metabolism. Sugar metabolism was diverted towards the production of α-acetolactate, the precursor of diacetyl, by either disruption of lactate dehydrogenase or by the nisin inducible expression system (NICE), through the overproduction of NADH oxidase. By combining the latter strategy with disruption of the gene encoding α-acetolactate decarboxylase, high diacetyl production from glucose and lactose was achieved. The production of this bacteriocin is strongly regulated through auto induction. In the absence of nisin, the nisin-biosynthetic genes are not expressed, and the degree of expression is directly proportional to the amount of inducer (nisin) added [94]. Using this system an efficient alanine-producer was constructed by introducing the gene coding for alanine dehydrogenase in the NICE system [94].

The development of new starter cultures for the production of fermented foods, that could meet the changing consumer preferences and expectations for safe products with specific characteristics, is studied through (a) the composition of mixed-strain cultures isolated from nature or (b) the genetic engineering of existing isolates. A variety of techniques involving natural selection and evolution are available to enhance the performance of existing strains, including the isolation of mutants with desired properties, adaptive laboratory evolution, genome shuffling, and genome editing [95],[96]. However, for food applications, recombinant DNA technology is strongly limited by regulations [95],[97],[98] and the negative consumer perspective towards GMOs [95]. Hence, this field mainly relies on classical untargeted and laborious methods based on natural selection and evolution, such as mutagenesis and adaptive laboratory evolution [96],[98],[99] which are considered non-GMO, while other non-targeted methods resulting in non-GMO strains are transduction and conjugation [100].

As already mentioned, LAB are so multifunctional and fulfill many important functions in foods, such as the improvement of overall quality of the food and contribution to the development of specific flavor, as well as the improvement of the safety by inhibiting spoilage and pathogenic microorganisms, and a number of research papers have been focused on the use of autochthonous starter cultures. That is, the use of multiple strain cultures which would be representative of the microbial composition of the food and would ensure high performance during the manufacture of fermented foods. Thus, the use of autochthonous starter cultures has been studied for a variety of fermented foods such as Mozzarella cheese [101], white pickled cheese [102], spreadable goat's cheese [103], Sucuk, a Turkish dry-fermented sausage [104] and Chorizo [105],[106].

## 5. Conclusions

LAB are the most commonly used microorganisms for the fermentation and preservation of foods. Their importance is associated mainly with their safe metabolic activity while growing in foods utilising available carbohydrates for the production of organic acids and other metabolites. Their common occurrence in foods, alongwith their long-lived uses, contributes to their natural acceptance as GRAS (Generally Recognised As Safe) for human consumption [107]. The EFSA's “Panel on Biological Hazards (BIOHAZ)” has concluded that the fermenting bacteria associated with food, whether resistant to antibiotics or not with the possible exception of enterococci do not pose a clinical problem [107]. However, they can act as a reservoir for transferable resistance genes. Strains with genes transferable in such a way could inter the food chain and increase the probability of a transfer to food associated intestinal pathogenic organisms.

The development of fermenting bacterial cultures was huge during the last 15 years, since the discovery of the complete genome sequence of *Lc. lactis* subsp. *lactis* IL1403 by Bolotin et al. in 2001 [4] and a variety of commercial starter, functional, bio-protective and probiotic cultures with desirable properties have marketed. Advances in the genetics, molecular biology, physiology, and biochemistry of LAB have provided new insights and applications for these bacteria. Food industry is now capable of producing safe and nutritious products with different flavours, sometimes with special health-promoting properties, which satisfy the demands of all consumer and market niches, and to resemble the characteristics of the traditional products. In addition, the use of selected strains of given species with known metabolic properties and high technological performances has improved the total quality control of the manufacturing process.
